# Sequential Slot-Die Deposition of Perovskite Solar Cells Using Dimethylsulfoxide Lead Iodide Ink

**DOI:** 10.3390/ma11112106

**Published:** 2018-10-26

**Authors:** Daniel Burkitt, Justin Searle, David A. Worsley, Trystan Watson

**Affiliations:** SPECIFIC, Swansea University, Swansea SA1 8EN, UK; daniel.burkitt@swansea.ac.uk (D.B.); j.r.searle@swansea.ac.uk (J.S.); D.a.worsley@swansea.ac.uk (D.A.W.)

**Keywords:** perovskite, slot die, sequential, dimethylsulfoxide, lead iodide, coating

## Abstract

This work demonstrates a sequential deposition of lead iodide followed by methylammonium iodide using the industrially compatible slot-die coating method that produces homogeneous pin-hole free films without the use of the highly toxic dimethylformamide. This is achieved through the careful selection and formulation of the solvent system and coating conditions for both the lead iodide layer and the methylammonium iodide coating. The solvent system choice is found to be critical to achieving good coating quality, conversion to the final perovskite and for the film morphology formed. A range of alcohols are assessed as solvent for methylammonium iodide formulations for use in slot-die coating. A dimethylsulfoxide solvent system for the lead iodide layer is shown which is significantly less toxic than the dimethylformamide solvent system commonly used for lead iodide deposition, which could find utility in high throughput manufacture of perovskite solar cells.

## 1. Introduction

Solar energy and photovoltaics are becoming an increasingly important part of the electrical power generation mix. Essential to reducing the costs of this energy source is the reduction in the cost of photovoltaic modules, many methods to accomplish this are being pursued such as alternative deposition methods for conventionally used silicon photovoltaic materials [1,2] and entirely new photovoltaic materials such as cadmium telluride, copper indium gallium selenide, copper zinc tin sulfide [3], organic photovoltaic materials [4] and perovskites. Perovskite photovoltaics have rapidly become the subject of intense research efforts due to their unique properties of potentially low materials costs [5,6,7], suitability for solution processing [8], intriguing device physics [9,10,11] and astonishingly high efficiencies [12,13]. These factors coupled with high throughput manufacturing techniques could lead to reduced production costs compared to other printed and coated photovoltaics, as well as to conventional photovoltaics.

The most commonly used deposition method for perovskite photovoltaics in laboratory research is spin coating. This is generally not seen as a viable technique for large scale manufacture due to limits on the size of coated areas (<1 m${}^{2}$), high material wastage and incompatibility with high throughput techniques such as roll-to-roll coating [14]. Many techniques have been proposed as more viable large scale manufacturing processes, these include printing techniques such as screen [15,16] and ink-jet [17,18,19] printing and coating techniques such as spray [20,21,22] and doctor-blade coating [23,24,25], as well as slot-die coating [26,27,28,29,30,31,32,33,34,35,36,37,38,39,40,41,42,43].

Slot-die coating stands out as particularly suitable as it can be used in both a continuous roll-to-roll process with flexible substrates or a batch process with rigid or flexible substrates. As a pre-metered coating method there is very little material wastage, adding to its cost effectiveness compared to spin coating. It also gives the ability to perform simple patterning such as stripes [44,45,46,47] or patches [48,49,50] of material, which could be advantageous when producing photovoltaic modules. The coating technique is also well suited to producing thin films of material, as used in perovskite photovoltaics, from a wide range of ink rheologies. The technique can achieve considerable line speeds and so help to improve throughput, slot-die coating has been successfully used for the production of flexible electronic devices, including structurally similar organic photovoltaics, at high throughput [51].

Photo-active lead-based perovskite materials are generally of the form ABX${}_{3}$, where A is a lead ion, B is a cation and X is an anion. The precursors for the perovskite ink are typically a lead salt, usually a lead halide such as lead iodide, providing the A site material. The B site cation is typically an alkyl-ammonium ion such as methylammonium or formamidinium or an inorganic ion such as caesium. These are typically introduced to the ink as a halide salt, e.g., methylammonium iodide (MAI) or caesium iodide. The X site anions are usually halides, that can be introduced through the counter ions of the lead salt and the cation precursors.

Achieving a uniform layer of the correct thickness of perovskite material in the device stack is a critical step to achieving high performance [52]. To harvest the maximum power from the device the layer must be free from defects that can result in shunt current leakages between the electrodes either side of the perovskite layer. A homogeneous deposition of a layer with optimised thickness maximises light absorption and promotes efficient charge collection and so minimises losses due recombination of photogenerated charges. The morphology and topography of the perovskite layer strongly influences the charge transport properties of the layer and the rate of recombination. Grain boundaries and other crystal defects can act as recombination centres and inhibit charge transfer through the layer and result in loss of performance. Ideally the grain size parallel to the substrate should be maximised, i.e., large planar grains to reduce the number of grain boundaries and give an optimal interface with the adjacent charge extraction layers, with a compact morphology with fused grain boundaries to avoid large voids and pin-holes between the grains that could increase shunt losses that could increase [53,54].

For solution processed perovskite solar cells the perovskite layer is typically deposited using one of two main deposition strategies. Either the perovskite precursors are mixed as a single ink and coated from this in a ‘single step’ process or the precursors are made into separate inks and a ‘sequential deposition’ process is used [55,56,57]. Typically for the sequential deposition process the lead salt (e.g., lead halide) is dissolved in a solvent such as dimethylformamide (DMF) or dimethylsulfoxide (DMSO), or with these and others e.g., gamma-butyrolactone in mixed solvent systems, then deposited from this ink to form a solid film of the lead halide. This film is then exposed to a formulation comprising the cation source (e.g., methylammonium halide) dissolved in a solvent, typically an alcohol, this step is commonly performed using dip coating or spin coating. The lead halide film reacts with the alkyl-ammonium halide and on drying crystallises to form the final organic–inorganic perovskite.

Although single step spin coating using a “solvent drip” method can result in perovskite layers with excellent film morphology [58] this is difficult to integrate into a slot-die coating process. For coating processes not requiring a “solvent drip” the sequential deposition process can generally achieve better film morphology and coverage than the single step methods and so can achieve higher performance, but has the disadvantage of requiring at least two coating steps. The sequential deposition process can also allow for interesting alternative methods for controlling the crystal structure and composition of the perovskite film, e.g., by seeding the initial layer with nucleation sites [59].

The use of slot-die coating in the fabrication of perovskite solar cells has already been demonstrated using both the single step and sequential deposition methods. Sequential deposition has been reported to require shorter drying times [28], whereas for the single step perovskite slot-die coatings reported using a lead chloride and MAI precursor formulation have required long drying times of 90 min [32,41], a hindrance to economical manufacture. Sequential deposition slot-die coating has been shown to be particularly sensitive to the deposition conditions and has required extra process steps such as enclosed chamber annealing [27] and mediator extraction treatment [42] to achieve a porous lead iodide film formation that can be readily converted to perovskite.

The perovskite most commonly used with slot-die coating has been the organic–inorganic material methylammonium lead triiodide. In most of these works DMF has been used as the main solvent for the perovskite inks, this would pose several challenges for use in large scale manufacture, not least the toxicity of the solvent.

The aim of this work is to develop a slot-die coating sequential deposition process for methylammonium lead triiodide perovskite that does not require the use of the toxic solvent DMF and to also develop a slot-die coating process which also removes any prohibitive extra stages, such as enclosed chamber storage, that would be difficult to integrate into a manufacturing process. To this end, the use of non-toxic DMSO as a replacement for DMF as the lead iodide solvent is demonstrated, which results in both improved coating quality and device performance compared to DMF.

**Figure 1:**
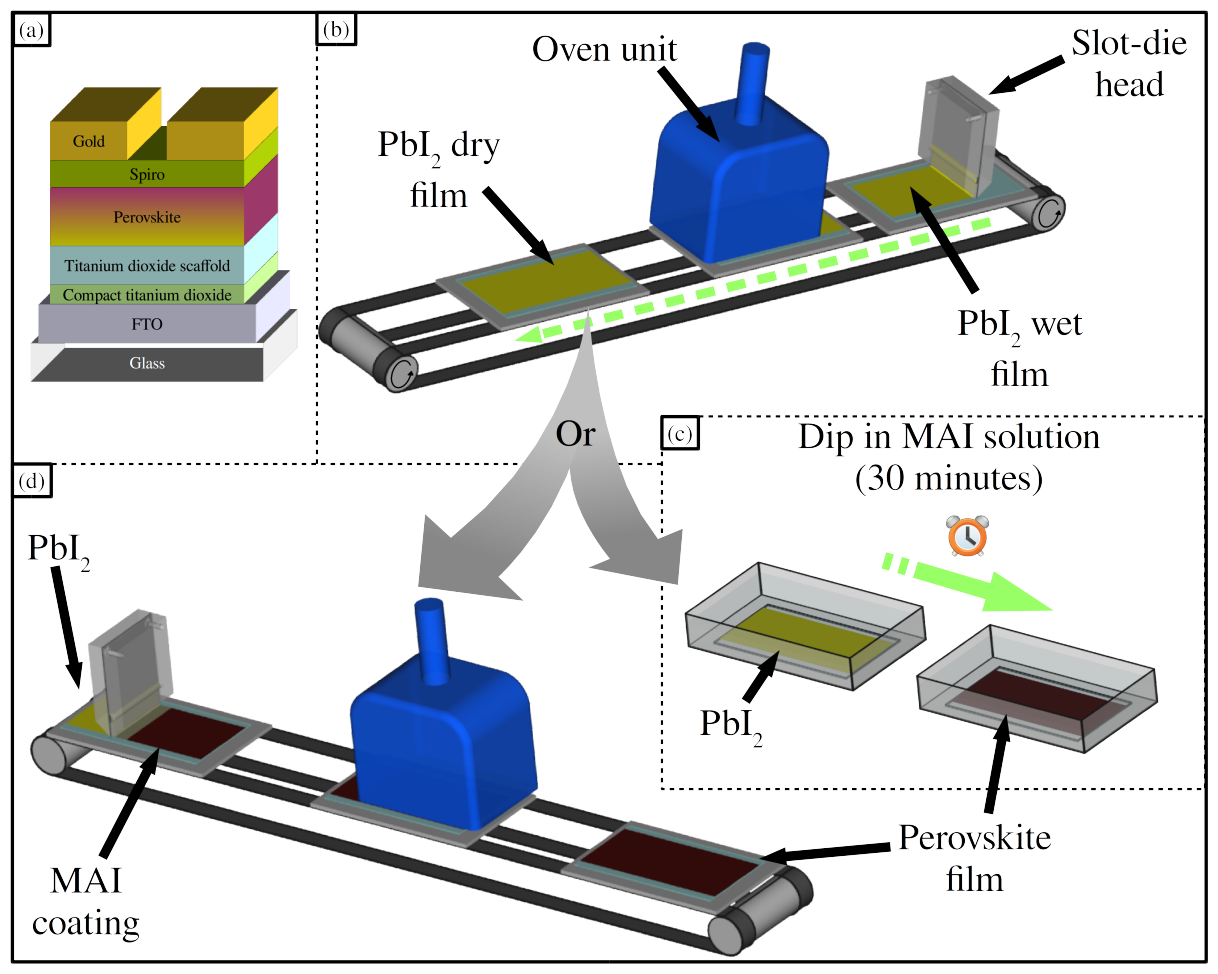
(**a**) The full device stack, (**b**) a schematic of lead iodide slot-die coating, (**c**) dip coating or (**d**) slot-die coating of MAI.

In addition, the alcohol used as solvent for the slot-die coated MAI ink is optimised using a room temperature process, with no additional treatments of the lead iodide layer. Further improvements in device efficiency are achieved using a heated substrate coating process for deposition of the lead iodide layer which results in a film more readily converted to perovskite using slot-die coating of the MAI ink and results in device performance equal to that of cells converted to perovskite using a 30 min dip coating step. Schematics of the coating processes used and the device stack produced are shown in Figure 1.

## 2. Results

### 2.1. Lead Iodide Layer Slot-Die Coating

Previous reports of slot-die coated lead iodide layers have made use of DMF as a solvent for formulations, this poses a challenge when moving to scaled-up production of perovskite solar cells on larger coating equipment as the solvent is highly toxic and would require complex expensive containment and extraction methods to safely use. DMSO has been utilised successfully as a solvent in many perovskite and perovskite precursor formulations and has successfully been used as an additive added in small quantities to single step formulations of perovskite inks for slot-die coating [30] or for formulations of lead iodide in a 1–9 ratio with DMF used for slot-die coating of the lead iodide layer in sequential deposition, resulting in high performance devices [42]. As well as being used as the sole solvent for slot-die coating lead iodide layers for use in sequential slot-die deposition (although not optimised for performance) [39], it also has the benefit of being non-toxic. DMF has a Time Weighted Average (TWA) Workplace Exposure Limit (WEL) of just 5 ppm (from materials Safety Data Sheet (SDS)), much lower than other common solvents e.g., ethanol WEL TWA (1000 ppm) (from SDS), in comparison DMSO is not a hazardous substance according to Regulation (EC) No. 1272/2008e (from SDS) and so no exposure limits have been set for it. Although solvent containment methods would still be required when using such formulations, due to any toxic components that might be transported by the solvent vapours, removing the toxicity of the main solvent in the formulation is still critical to achieving an ink that can be used safely in a range of coating environments. Given this, the use of DMSO as the sole solvent for slot-die coating formulations of lead iodide is preferred and is the core development presented here.

The device structure used in this work includes a mesoporous titanium dioxide scaffold on to which the lead iodide layer is coated, this layer needs to fully and uniformly infiltrate the scaffold and to form a capping layer over the mesoporous titanium dioxide surface. As well as this, as noted in other works, to convert to perovskite efficiently the lead iodide must not form a too compact layer such that the subsequent MAI coating can not easily penetrate and convert the layer to perovskite [27,42]. The morphology of the lead iodide layer formed will directly impact on the final perovskite morphology once converted, e.g., by the number of initial nucleation sites provided [60]. To achieve adequate infiltration of the mesoporous layer it is necessary for any formulation to wet well on the surface of the layer.

The lead iodide ink for slot-die coating was formulated in solvent systems of either DMF or DMSO with solids content of 20 wt%. To determine wetting behaviour the contact angle of the DMSO formulation on a mesoporous titanium dioxide layer, as well as on the blocking layer, was measured and compared to that of the DMF formulation. The contact angles measured are summarised in Table 1, the DMF-based ink was found to have a viscosity of 0.89 mPa·s compared to 2.54 mPa·s for the DMSO-based ink and surface tension of 35.3 m·Nm${}^{-1}$ compared to 40.3 m·Nm${}^{-1}$ for the DMSO-based ink. On the mesoporous surface the DMSO ink shows a higher initial contact angle than the DMF-based ink but both inks fully wet the surface over time, with static contact angles of <5${}^{\circ }$, with the DMSO-based ink wetting at a slower rate taking over 5 s to fully wet compared to around 0.5 s for the DMF-based ink. On the compact blocking layer surface the DMSO-based ink shows a relatively high static contact angle of 22.5${}^{\circ }$ whereas the DMF-based ink shows complete wetting on the surface. The mesoporous layer was coated on the substrates as two stripes, with an underlying layer of spray coated blocking layer covering the entire substrate. The differences in contact angle of the two inks might be expected to result in different film qualities, as the initially coated wet film will spread depending on the relative wetting of the ink on the substrate.

**Figure 2:**
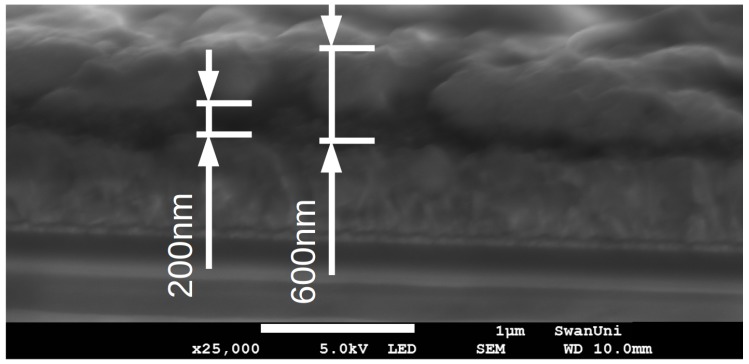
SEM cross section image of slot-die coated lead iodide film, converted to perovskite, on an FTO coated glass substrate with titanium dioxide blocking layer and scaffold. The approximately 200 nm scaffold and 600 nm perovskite layer are marked in the image, the scale-bar in the boarder represents 1 micron.

To further investigate this, both lead iodide formulations were slot-die coated from an approximately 10 μm lead iodide wet film thickness, to give films that once converted with MAI, would form approximately 600 nm thick perovskite dry films, with approximately 200 nm incorporated in the scaffold layer and 400 nm as a capping layer over the scaffold, as shown in Figure 2. The coating machine has the substrate held on a platen that travels on a belt that moves the platen and substrate under the coating head and on into the oven unit. Coatings were made at 1 m·min${}^{-1}$ followed by directly travelling, over a distance of 30 cm, into the coater oven unit to be dried at approximately 105 ${}^{\circ }$C over a distance of approximately 30 cm with a line speed of 0.1 m·min${}^{-1}$, to remove excess solvent, before being moved in to a fan oven and dried for a further 7 min at 100 ${}^{\circ }$C.

**Figure 3:**
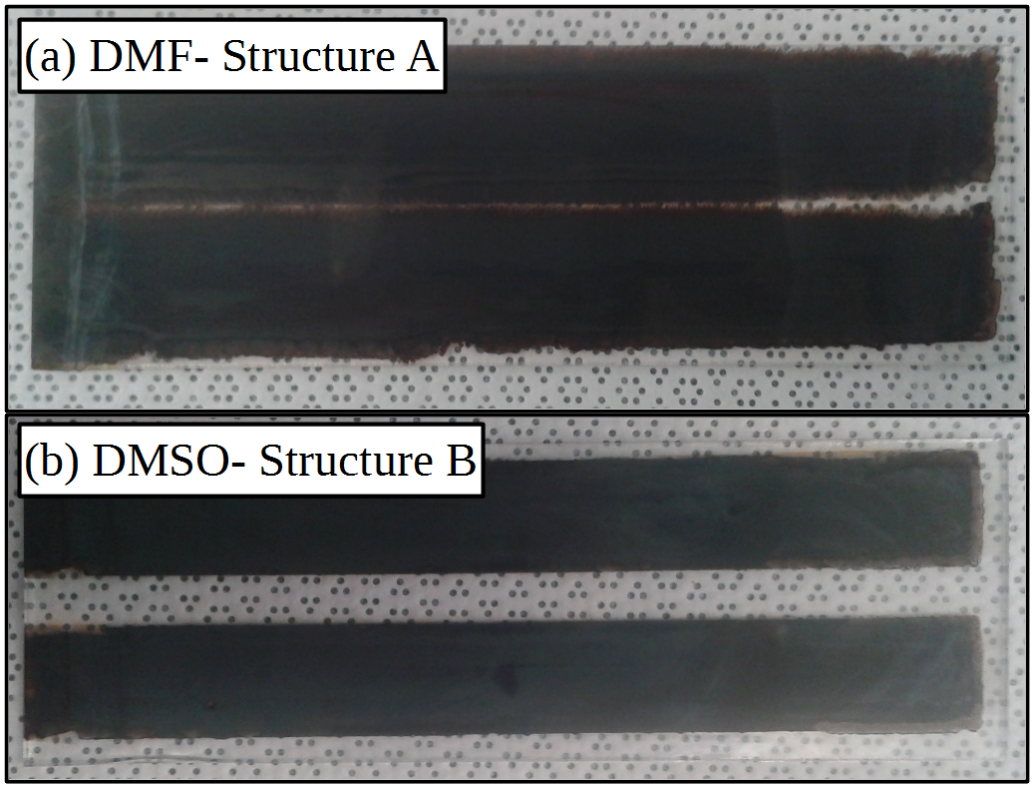
Pictures of perovskite films formed from coatings of lead iodide from either (**a**) DMF or (**b**) DMSO-based inks. Substrates are 15 cm by 5 cm.

The difference in rheology of the two inks result in markedly different film formation, images of both slot-die coating types which were then subsequently converted to perovskite by dip coating (to give improved contrast in images) are shown in Figure 3, for the lead iodide films both dried at 105 ${}^{\circ }$C. The film produced using a DMF-based ink has over wet across the substrate and the stripe definition has been lost. This would be expected to be detrimental to device performance as the wet film thickness will vary and so the dry film thickness will no longer be as expected and will vary across the coating. The film produced using a DMSO-based ink shows the formation of the expected two stripe pattern with no over wetting, showing the improved film formation, compared to the DMF ink, under these coating and drying conditions. The improved film formation of the DMSO ink over the DMF ink can be explained by the poor wetting of the DMSO ink on the compact titanium dioxide surface that acts to contain the ink on the initially coated areas with underlying mesoporous scaffold, as well as by the relatively slower wetting of the DMSO-based ink on the mesoporous scaffold compared the DMF-based ink. Both films are continuous and do not show signs of coating defects such as ribbing or break up of the coating bead that would indicate a low flow limit being reached, this shows that despite the higher capillary number [61] of the DMSO-based ink it is still suitable for slot-die coating under these coating conditions.

**Figure 4:**
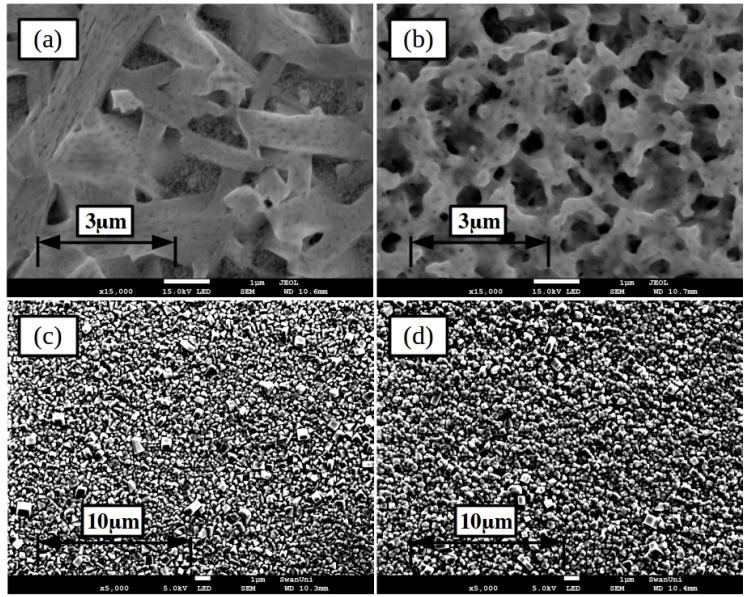
SEM images of lead iodide films formed from coatings of formulations based on either (**a**) DMF or (**b**) DMSO and once converted to perovskite by dip coating, (**c**) DMF and (**d**) DMSO. Inset scale-bar lengths are given in inset labels.

The morphology of the lead iodide layers was studied using scanning electron microscopy (SEM), images of the slot-die coated films, from either DMF or DMSO are given in Figure 4. The film formed from the DMF-based formulation shows large needle like crystal grains, of the order of several microns in length, growing from the mesoporous titanium dioxide layer with voids between where the mesoporous layer is still visible. In comparison the film formed from the DMSO-based ink shows a more porous structure made up of smaller grains, with diameter in the order of a micron or less, there is also much less of the mesoporous scaffold visible. The morphology of the lead iodide film from the DMSO ink would be expected to react with MAI and convert to perovskite more rapidly than the DMF ink films due to the increased surface area of the smaller grains giving more reaction sites as well as the porous structure allowing the formulation to penetrate the film more easily, so speeding the reaction and conversion.

### 2.2. Methylammonium Iodide Slot-Die Coating

Having produced lead iodide films via slot-die coating the conversion to perovskite was investigated. The most commonly used method to convert lead iodide to the final perovskite is through the use of dip coating, normally by placing the lead iodide film in a dilute solution of MAI in 2-propanol (IPA), often for extended periods of time from a few minutes to tens of minutes. Slot-die coating of MAI formulations directly on to the lead iodide film has also been reported [27,28,39], as the solvent rapidly evaporates any reaction to form perovskite occurs within a much shorter time period than for most dip coatings so achieving complete conversion to perovskite in this time frame is challenging. Heating of the substrate has been reported to improve the conversion to perovskite [27] by accelerating the reaction (but also speeds evaporation of solvent), but this adds extra complications to the coating process so ideally a coating method avoiding this would be preferred.

To determine the conversion to perovskite and to test and compare the performance of devices, incorporating the slot-die coated DMF and DMSO-based lead iodide films, dip coating was used initially. The films were placed in a solution of MAI for 30 min, a method previously found to give good conversion to perovskite.

**Figure 5:**
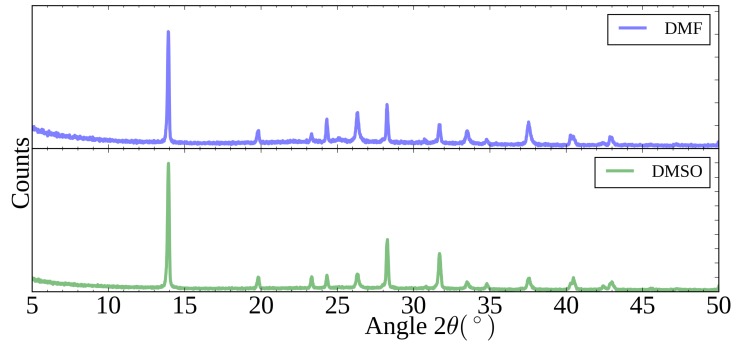
XRD spectra of perovskite films formed from dip coating of lead iodide films from either DMF or DMSO-based inks.

X-ray diffraction spectra of the perovskites formed from both films are given in Figure 5, in both cases the expected peaks for the perovskite structure are present, with the peak at 14.1${}^{\circ }$ dominant, representing the 110 reflection, for both films there is no signal for lead iodide at around 12.7${}^{\circ }$. This indicates good conversion to the final perovskite, using dip coating, for films produced from both lead iodide inks.

As discussed previously in the context of wetting, SEM images of the perovskite formed are given in Figure 4, in both cases very similar structures are formed with excellent coverage over the underlying mesoporous layer and crystal grains of around 1 micron diameter formed.

**Figure 6:**
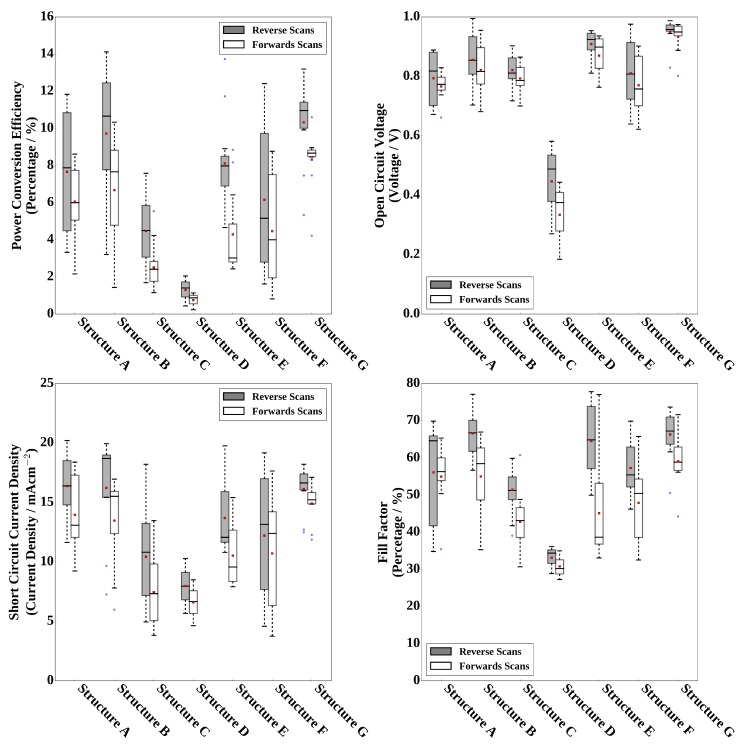
Box-plots of JV scan photovoltaic parameters for 0.09 cm${}^{2}$ cells, see table for description of device structures. The boxes represent the first and third quartiles, the horizontal black line the median, the upper whisker the data within 1.5 times the inter quartile range of the upper quartile and the lower whisker 1.5 times the inter quartile range of the lower quartile, red square the mean and blue dots outliers.

Table 2 gives the median current-density voltage (JV) curve parameters of devices masked to 0.09 cm${}^{-1}$ made using the DMF (structure A) or DMSO (structure B) lead iodide films converted using dip coating, in both cases high efficiency devices are produced, results are also summarised in Figure 6 as box-plots. The DMSO-based devices show greater power conversion efficiency (PCE) than the DMF-based devices, this is mostly attributed to an improved light shunt resistance (Rsh), of 1249 compared to 1017 ohms·cm${}^{2}$ (taken from the reverse light JV scan curve), so improved fill factor (FF), open circuit voltage (Voc) and short-circuit current-density (Jsc). This could be attributed to the better stripe coating quality and more uniform lead iodide coating of the DMSO-based ink that gives a more optimised perovskite film thickness that results in greater charge carrier generation and improved shunt resistance and fewer losses of charge carriers to recombination.

Having established that the DMSO-based lead iodide films could form good quality perovskite layers and high performance devices the use of slot-die coating, rather than the conventional dip coating, for the deposition of the MAI ink was investigated. To achieve complete conversion of lead iodide to perovskite the MAI formulation would have to sufficiently wet and infiltrate into the lead iodide film to bring the two components in contact with each other and to react and form perovskite [62,63]. For the dip coating process the use of other solvents has been investigated and in some cases found to improve conversion to perovskite, ethanol has been shown to be superior to IPA for the dip coating process, where it was suggested the lower viscosity of ethanol improved the kinetics of the reaction [64].

As well as converting to a high degree the morphology of the resulting perovskite film is critical to device performance and the carrier solvent for MAI will strongly influence the morphology formed. Alcohol solvents with various structures have been shown to influence the morphology of sequentially grown perovskite crystals, in particular a dissolution–recrystallisation process (Ostwald ripening effect) dependant on the molecular polarity of the solvent has been suggested to be influential [65]. The static relative permittivity, used as a macroscopic measure of the polarity of the solvent, has also been highlighted as important for sequentially deposited perovskite crystal growth, the size of grains and the number of grain boundaries formed in films, with a higher permittivity facilitating the formation of films with larger grains and fewer grain boundaries [66].

As well as this, the rheology of the solvent and in particular viscosity will determine how rapidly the formulation infiltrates the porous lead iodide film [67]. For slot-die coating the volatility of the solvent is also expected to be critical, as the ink wet film is generally only a few tens of microns and so solvent will quickly evaporate giving a short time frame for both the formulation to infiltrate the film and for the reaction to occur [68]. The solubility of MAI in the solvent will also be important as MAI will rapidly crystallise out of solution from weak solvents as the solvent evaporates. The MAI will no longer be available to react, potentially crystallising on the surface of the film resulting in a poor interface with the hole transport layer deposited on top of it [69] and forming large crystallites providing a pathway for degradation reactions, for instance with the top electrode or via atmospheric moisture. The overall macroscopic coating quality of the layer including film defects such as discontinuity will depend on the rheology of the formulations and the particular coating conditions.

In order to investigate the role the solvent in conversion to perovskite for slot-die coating, formulations of MAI in several different alcohols, with various rheologies and volatilities, were prepared, these are listed in Table 3. Methanol has a low viscosity but is also very volatile, ethanol has a slightly higher viscosity and is less volatile, IPA (the solvent used for most slot-die sequential depositions reported) is of similar volatility to ethanol but has a higher viscosity and 1-butanol has a higher viscosity still and is less volatile.

It should be noted that the concentration of MAI in the formulation can also alter the crystallisation process and the morphology of perovskite films formed [70]. To control for this the concentration of MAI in the formulations was kept constant and was chosen as a balance of being accessible within the solubility limits of the solvents and not requiring an excessively great wet film thickness to achieve a 1:1 molar ratio of MAI and lead iodide. Too great a wet film thickness could cause non-uniform drying or cause a build up of ink at the upstream lip of the coating head e.g., flooding/dripping and a loss of pre-metering [71] when coating at too low a speed.

All the MAI formulations showed complete wetting on the lead iodide surface with static contact angles less than 5${}^{\circ }$. The MAI formulations were slot-die coated onto lead iodide coated substrates in order to establish the conversion to perovskite for each ink and the morphology of the layer formed. For all the formulations the coating and drying conditions were kept constant, with a coating speed of 1 m·min${}^{-1}$, followed by allowing excess solvent to evaporate at room temperature and then drying in a fan oven at 90 ${}^{\circ }$C.

**Figure 7:**
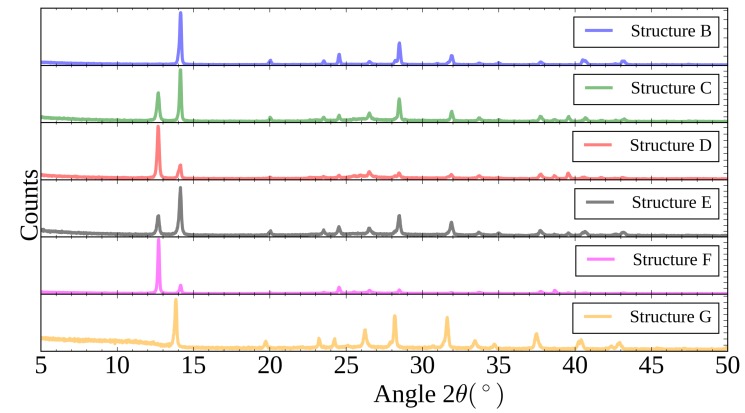
XRD spectra of perovskite films formed from (structure B) dip coating or (structures C to G) slot-die coating of methylammonium iodide alcohol-based formulations on lead iodide films.

Figure 7 shows the XRD spectra taken for each of the films, clearly the level of conversion to perovskite is low for the methanol (structure D) and 1-butanol (structure F)-based formulations, indicated by the large peak at 12.7${}^{\circ }$ corresponding to residual lead iodide. The poor conversion for the methanol-based ink films can be linked to the high volatility of the solvent and the rapid evaporation of it from the film resulting in there being little time for more extensive conversion to perovskite. The poor conversion of the 1-butanol ink films can be linked to the rheology of the ink with the higher viscosity slowing the rate of reaction and penetration of solution into the lead iodide film. The IPA (structure C) and ethanol (structure E)-based formulations show slightly greater conversion to perovskite, with the ethanol slightly greater, but not complete conversion as seen for the dip coated films (structure B).

**Figure 8:**
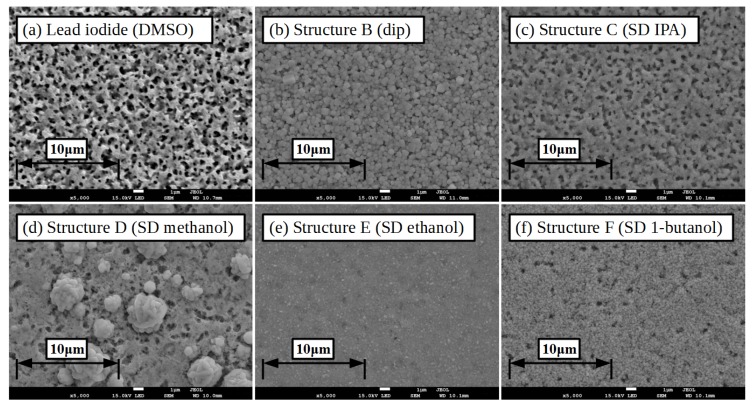
SEM images of initial lead iodide film and perovskite films formed from coating methylammonium iodide on lead iodide coated substrates. Inset scale-bar lengths are given in inset labels. (**a**) Initial lead iodide slot-die coated on room temperature substrate film from DMSO, (**b**) with MAI dip coated, (**c**) with MAI slot-die coated from either IPA, (**d**) methanol, (**e**) ethanol, or (**f**) 1-butanol.

Figure 8 shows SEM images of the films formed from each formulation, the methanol-based formulation films show what appear to be large perovskite crystals growing out of a bed of lead iodide with very limited and sporadic conversion to perovskite. The high volatility of the solvent and the rapid evaporation of it from the film results in little time for conversion to perovskite to take place and this is localised to small areas. The film also appears to have lost some of the initial lead iodide film structure with the lead iodide recrystallising to a more planar structure, which might be due to the high relative permittivity of methanol facilitating the dissolution–recrystallisation process. The IPA based formulation results in films with loosely packed crystal grains of the order of hundreds of nanometres in diameter with a general structure similar to that of the initial lead iodide film. Whereas the ethanol-based formulation shows smaller more densely packed crystals, where the surface is much more uniformly structured, possibly due to the higher static relative permittivity of the solvent increasing the dissolution-crystallisation of the initial film and loss of initial structure. The 1-butanol formulation results in small grains but more loosely packed than the ethanol formulation, Clearly a balance of volatility, rheology, solubility of MAI and permittivity of the alcohols plays a crucial role in the conversion to perovskite and the morphology formed.

Photovoltaic devices were fabricated using the different slot-die coated MAI films, Table 2 and Figure 6 summarise the JV scan photovoltaic parameters measured for these. Clearly the methanol-based formulation (structure D) results in poor device performance, as would be expected from the poor conversion to perovskite. The devices produced from the IPA and 1-butanol-based formulations (structure C and F) showed some cells with better efficiencies than those made using the methanol formulation, but not as good as for dip coated films, with a low short-circuit current density being the main cause of poor performance. The 1-butanol-based formulation devices also show a large spread in device performances, despite the very poor overall level of conversion inferred from the XRD results this suggests some areas of more optimal conversion to perovskite and some less. The ethanol (structures E) devices showed some very encouraging performances, with power conversion efficiencies approaching that of the dip coated cells but with a wide spread in the performances measured. This despite the relatively poor level of conversion to perovskite, but excess lead iodide even to levels of 20% or more has been shown to result in working devices [69]. The IPA, methanol and 1-butanol-based slot-die MAI ink devices suffered from increased series resistance (taken from the reverse scan light JV curve) compared to the dip coated films, of 10.8, 20.1 and 9.3 ohms·cm${}^{2}$ respectively compared to 7.0 ohms·cm${}^{2}$ for the dip coated films and 6.6 ohms·cm${}^{2}$ for the ethanol-based slot-die ink, which could be due to the lower conversion to perovskite and residual resistive lead iodide. All of the devices show significant hysteresis in the JV curves between reverse and forward scans.

**Figure 9:**
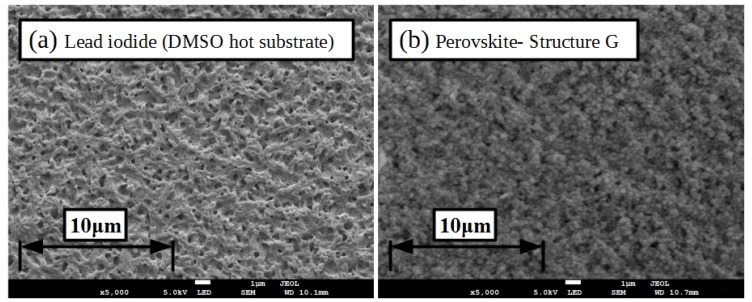
SEM images of (**a**) lead iodide film from coating on 100 ${}^{\circ }$C substrates and (**b**) the perovskite film formed from coating MAI in ethanol on the resulting film. Inset scale-bar lengths are given in inset labels.

In an attempt to further improve the device performance a modified lead iodide coating process was developed to produce more labile lead iodide films in order to increase conversion to perovskite when slot-die coated with the MAI in ethanol ink. The substrate was pre-heated to 100 ${}^{\circ }$C and then the lead iodide ink coated on to the hot substrate. This resulted in lead iodide films that were found to convert to perovskite almost fully when MAI in ethanol was slot-die coated over them, as shown in the XRD spectra in Figure 7 (structure G). The surface morphology of the resulting films is shown in the SEM images in Figure 9, the surface shows more undulations than that produced on room temperature substrate coated lead iodide films, with more small areas of the mesoporous scaffold visible, but still with good overall coverage of the scaffold. Comparing the perovskite films formed from the room temperature and heated substrate lead iodide films with slot-die coated MAI from the ethanol formulation the grain size is increased for the heated substrate, but the film is less planar and seems to retain more of the structure of the initial lead iodide film.

Performance of devices made using these films are given in Table 2 and Figure 6, (structure G). The average PCE achieved is significantly improved compared to the cells with lead iodide coated at room temperature and slot-die coated MAI in ethanol formulation (structure E). This is mainly due to an increase in Jsc, which can be related to the improved conversion to perovskite resulting in more material contributing to photo-current generation. There are also smaller improvements in Voc and FF that also contribute to the overall improved median PCE of 11.0% compared to 8.0% for structure E, which is comparable to the efficiency (10.7%) of the dip coated devices (structure B). The shelf-life stability of devices using slot-die coated films of lead iodide coated on heated substrate with either dip coated or slot-die coated MAI are compared. Devices were encapsulated with Kapton tape, UV curable adhesive and a glass coverslip and stored in the dark in a sealed box containing desiccant, devices were then tested again after 442 days. The slot-die coated devices showed similar levels of degradation to the dip coated devices and retained approximately 60–70% of the original efficiency values, in line with what is usually seen for this device structure and perovskite type [32,72], with an increase in series resistance and decrease in Jsc, but little change in FF, as shown in Table 4. This result suggests that the slot-die coating process developed for MAI is not detrimental to shelf-life stability compared to dip coating.

## 3. Materials and Methods

### 3.1. Device Fabrication Methods

Device fabrication was performed in a humidity controlled (30% relative humidity) clean-room (class 7) environment. Devices were fabricated on fluorine doped tin oxide (FTO) coated glass substrates with a device stack of spray coated compact titanium dioxide blocking layer, slot-die coated mesoporous titanium dioxide scaffold, slot die coated lead iodide layer converted to perovskite using methylammonium iodide by either dip or slot die coating followed by spin coated spiro-MeOTAD hole transport material and evaporated gold top contacts.

FTO glass (TEC8) substrates were etched using tape to define electrode areas, zinc powder and hydrochloric acid to remove FTO and rinsed with deionised water then cut into 15 cm by 5 cm sections. Substrates were further cleaned sequentially with acetone, dilute Hellmanex, deionised water and 2-propanol (isopropyl alcohol, IPA).

The compact titanium dioxide blocking layer formulation was made up by dilution of a solution of titanium diisopropoxide bis(acetylacetonate) (Sigma-Aldrich) in IPA. The layer was spray coated on substrates held at 300 ${}^{\circ }$C then cooled to room temperature before deposition of the mesoporous scaffold.

A bench-top slot-die coating unit, with in-line forced hot air oven, positioned in a fume-hood was used for coating. The slot-die coating head had a coating width up to 100 mm, patterned shims were used to define coated areas and corresponding meniscus guides [47] were used with the slot-die head to help form the meniscus between the slot-die head lips and substrate, the meniscus guide tab length was 1 mm. The gap height between the meniscus guide and substrate was set using feeler gauges and micrometer adjustment screws and set to as close to 50 μm as possible for each coating.

The mesoporous titanium dioxide layer was deposited using slot-die coating, the formulation was made up by dilution of DSL-18NRT titanium dioxide paste (Dyesol) with 1-butanol [41]. The formulation was stirred constantly to avoid agglomerations and filtered using 0.45 μm pore size regenerated cellulose syringe filters directly before use. The slot-die coated layer was deposited at a coating speed of 0.1 m·min${}^{-1}$ and a pump rate of 0.035 mL·min${}^{-1}$ over a coating width of 3.5 cm made up of two 1.75 cm stripes, giving an approximately 10 μm wet film thickness. The layer was dried on a hot plate at 140 ${}^{\circ }$C (10 min) to remove residual solvent then 325 ${}^{\circ }$C (10 min) to burn off organic materials followed by a 550 ${}^{\circ }$C sinter for 30 min.

The lead iodide layer was deposited by slot-die coating from a formulation of lead iodide (Sigma Aldrich 99%) 20 wt% in either DMF or DMSO (Sigma Aldrich, Anhydrous). The slot-die coated films were coated in air at 1.0 m·min${}^{-1}$ with a pump rate of 0.35 mL·min${}^{-1}$, over a width of 3.5 cm made up of two 1.75 cm stripes. Then directly dried in the coating machines in-line oven unit with an oven temperature of approximately 105 ${}^{\circ }$C (verified with thermocouples attached to substrate) over a distance of approximately 30 cm with a line speed of 0.1 m·min${}^{-1}$, to remove excess solvent, before being moved in to a fan oven and dried for a further 7 min at 100 ${}^{\circ }$C.

Formation of perovskite was achieved by either dip coating or slot-die coating with solutions of methylammonium iodide (MAI) (Dyesol). For dip coating the lead iodide films were submerged in a tray of MAI 10 mg·mL${}^{-1}$ in IPA for 30 min, followed by drying at room temperature and then carefully being rinsed with IPA from a wash bottle, then drying again at room temperature and then dried in a fan oven at 90 ${}^{\circ }$C for 10 min. Solutions for slot-die coated MAI were made up with IPA, methanol, ethanol or 1-butanol at 35 mg·mL${}^{-1}$ and coated at a speed of 1 m·min${}^{-1}$ over a width of 3.5 cm with a pump rate of 0.7 mL·min${}^{-1}$, giving an approximately 20 μm wet film thickness. The films were then allowed to dry at room temperature to remove excess solvent followed by drying in a fan oven at 90 ${}^{\circ }$C for 8 min then allowed to cool to room temperature before being carefully rinsed with IPA, followed by drying at room temperature then drying again at 90 ${}^{\circ }$C in a fan oven for 2 min.

The hole transport material spiro-MeOTAD (75 mg·mL${}^{-1}$ in chlorobenzene, with 10 μL 4-tert-Butylpyridine and 20 μL 600 millimolar Bis(trifluoromethane)sulfonimide lithium salt in acetonitrile per millilitre of solution) was spin coated at 2000 RPM for 45 s in a nitrogen filled glove-box, films were left overnight in air in a sealed box with desiccant to promote oxidation of the film [73,74].

Top contacts of gold were prepared by thermal evaporation under vacuum, using a shadow mask to define pixel areas. Conductive silver paste was applied to pixel contact areas to improve contact with pins on the device testing jig.

Cells for shelf-life tests were encapsulated using a UV curable adhesive (Ossila) and glass cover slip with a piece of Kapton tape to protect the hole transport material and perovskite layer from the adhesive before curing, the adhesive was cured by placing under a solar simulator for 10 min. The devices were stored in the dark in a sealed box with desiccant.

### 3.2. Test Methods

Current-voltage testing of devices was performed using a Keithley 2400 source measure unit and an Oriel solar simulator as light source (class AAA Newport Oriel Sol3A) calibrated to AM1.5 one sun equivalent intensity using a reference cell fitted with a KG5 filter (Newport Oriel 91150-KG5). Cells were masked to 0.09 cm${}^{2}$ for photovoltaic testing. Current-voltage curves were collected for both reverse and forwards sweep directions between 1.1 and −0.1V at a scan rate of approximately 0.15 Vs${}^{-1}$, in two-wire mode, 5 s of light soaking at open circuit was applied to the cell before scanning.

Viscosity measurements were made using a Malvern Bohlin Gemini 200 Nano HR rheometer, equipped with a temperature controlled stage, across a range of shear rates, all inks were found to be Newtonian at the shear rates used and viscosity is quoted as the value found for a shear rate of 25${}^{-1}$. Surface tension measurements were made using a FTA32 system and Pendant Drop Shape fitting routine. Scanning Electron Microscopy (SEM) images were collected on Jeol JSM-7800F field emission gun electron microscope, adjusting the working distance around 10 mm and using acceleration voltages of 5 or 15 kV. X-ray diffraction (XRD) spectra were collected using a Bruker D8 Discover instrument with a CuKα beam at 40 kV and 40 mA.

## 4. Conclusions

This work demonstrates that for sequential deposition of the perovskite layer by slot-die coating the choice of solvent and coating conditions is critical. For the lead iodide layer the coating quality and device performance can be improved by changing the solvent system. As such, the use of the non-toxic solvent DMSO is demonstrated and leads to high performance devices, this is important in the development of safe and cost effective scalable manufacturing processes for perovskite solar cells.

The choice of solvent system for the coating of the MAI layer has been shown to be critical to achieving conversion of the lead iodide layer to perovskite and achieving high performance. The use of ethanol as the solvent system for slot-die coated layers leads to devices with performance approaching those produced using the conventional dip coating method. Combined with an improved lead iodide coating method using a substrate pre-heated to 100 ${}^{\circ }$C, which results in lead iodide films more readily converted to perovskite when slot-die coated with MAI, devices with both lead iodide and MAI slot-die coated have been shown to achieve performance equivalent to that with dip coated MAI.

Further improvements to the quality of the perovskite layer will be necessary to achieve higher performance similar to that of spin coated devices and moving towards a viable manufacturing process. Improving the grain size and reducing grain boundaries of the perovskite would likely help this [66] and further optimisation of the MAI (or alternative cation) formulation and coating conditions is a potentially interesting area of research for achieving this. When transitioning the process to a roll-to-roll coating setting the low volatility of DMSO is a possible hindrance to being able to coat at high line speeds without requiring long oven path lengths, so designing formulations for the sequential deposition process that dry more rapidly (but maintain the low toxicity of the solvent system) or developing other rapid drying techniques is of considerable importance.

## Figures and Tables

**Table 1:** Static contact angles measured on mesoporous titanium dioxide or compact blocking layer of lead iodide inks.

Solvent	Contact Angle	Contact Angle
	Mesoporous Surface	Blocking Layer Surface
	(Degrees/${}^{\circ }$)	(Degrees/${}^{\circ }$)
	Instant/Static	Instant/Static
DMF	9.9/<5${}^{\circ }$	16.5/<5${}^{\circ }$
DMSO	20.0/<5${}^{\circ }$	23.0/22.5${}^{\circ }$

**Table 2:** Device structures, layer deposition methods and median device performance for 0.09 cm${}^{2}$ active area cells.

Structure	Solvent	Coating Method	Solvent	Scan Direction	Voc	Jsc	FF	PCE	Hero PCE
	Lead Iodide	MAI	MAI		(V)	mA·cm${}^{2}$	(%)	(%)	(%)
A	DMF	Dip	IPA	Reverse	0.82	16.4	64.6	7.9	11.8
				Forwards	0.77	13.1	56.4	6.0	8.6
B	DMSO	Dip	IPA	Reverse	0.86	18.7	66.7	10.7	14.1
				Forwards	0.82	15.5	58.5	7.7	10.3
C	DMSO	Slot-die	IPA	Reverse	0.81	10.8	51.3	4.5	7.6
				Forwards	0.79	7.3	43.2	2.5	5.5
D	DMSO	Slot-die	Methanol	Reverse	0.49	8.0	34.4	1.4	2.1
				Forwards	0.38	6.7	30.2	0.9	1.1
E	DMSO	Slot-die	Ethanol	Reverse	0.93	12.1	64.9	8.0	13.7
				Forwards	0.90	9.6	38.7	3.0	8.9
F	DMSO	Slot-die	1-Butanol	Reverse	0.81	13.2	55.5	5.2	12.4
				Forwards	0.76	12.4	50.5	4.0	8.8
G	DMSO	Slot-die	Ethanol	Reverse	0.96	16.7	67.2	11.0	13.2
	100 ${}^{\circ }$C substrate			Forwards	0.95	15.2	58.9	8.7	10.6

**Table 3:** Rheology measurements of MAI in alcohol formulations for slot-die coating.

Formulation Solvent	Viscosity at 25 s${}^{-1}$	Surface Tension
	(mPa·s)	(m·Nm${}^{-1}$)
Methanol	0.44	21.52
Ethanol	1.00	20.13
IPA	1.69	19.52
1-butanol	2.66	23.23

**Table 4:** Shelf-life stability of devices stored for 442 days, with slot-die coated lead iodide coated on heated substrates with either dip or slot-die coated MAI. Results are the mean of reverse direction light scans of devices, one device with three pixels was tested for each split, for the dip coated devices data for only one pixel is shown as the silver paste contacts to the other pixels had flaked away and good contact to the pixels could not be made.

MAI Coating Method	Dip		Slot-die	
	Structure B		Structure G	
**Parameter**	**Day 1**	**Day 442**	**Day 1**	**Day 442**
PCE (%)	12.0	7.2	11.3	8.3
Voc (V)	0.95	0.95	0.96	0.93
Jsc (mAcm${}^{-2}$)	16.8	12.2	17.2	13.5
FF (%)	75	62	68	66
Rs (Ohms·cm${}^{2}$)	5.1	6.3	5.0	8.7
